# Bullous Mastocytosis: A Rare Variant of Diffuse Cutaneous Mastocytosis

**DOI:** 10.7759/cureus.51660

**Published:** 2024-01-04

**Authors:** Shamma Khamis Almheiri, Jaheersha Pakran, Amani Abdulla AlFalasi, Reem El Bahtimi

**Affiliations:** 1 Dermatology, Dubai Academic Health Corporation (Dubai Health), Dubai, ARE; 2 Dermatopathology, International Dermpath Consult, Dubai, ARE

**Keywords:** mast cell, mast cell disorder, bullous dermatosis, pediatric dermatology, dermatology, bullous mastocytosis, diffuse cutaneous mastocytosis, mastocytosis

## Abstract

Mastocytosis is a disease of the mast cells caused by an increase in the number of mast cells due to abnormal proliferation. The disease is associated with a mutation in the *c-kit* gene, which is a key factor in the development of mast cells. Mastocytosis is classified into two main groups, namely, cutaneous and systemic mastocytosis, based on the site of mast cell accumulation. In cutaneous mastocytosis, the cells purely gather in the skin. In contrast, systemic mastocytosis must affect an internal organ, including the bone marrow, lymph nodes, liver, spleen, and/or the gastrointestinal tract with or without skin involvement. Cutaneous mastocytosis has four distinct presentations, including urticaria pigmentosa, cutaneous mastocytoma, diffuse cutaneous mastocytosis, and telangiectasia macularis eruptive perstans listed from most to least common. This case report presents a rare bullous variant of diffuse cutaneous mastocytosis.

## Introduction

Mastocytosis is a mast cell disease. An abnormal proliferation mediated by a mutated *c-kit* gene causes an increase in the number of mast cells [1-5]. The prevalence has been estimated to be 0.0001% of the general populace [2]. It does not exhibit any predilection toward a specific race [1,4]. Although the disorder can affect children and adults, the pediatric age group commonly presents with cutaneous mastocytosis, while the adult age group presents with systemic mastocytosis [1,2]. The classification of cutaneous mastocytosis and systemic mastocytosis is based on the site of mast cell accumulation. In cutaneous mastocytosis, the cells purely gather in the skin. In contrast, in systemic mastocytosis, cells must affect an internal organ, including the bone marrow, lymph nodes, liver, spleen, and/or the gastrointestinal tract with or without skin involvement [1-4]. Cutaneous mastocytosis has four distinct presentations, including urticaria pigmentosa, cutaneous mastocytomas, diffuse cutaneous mastocytosis, and telangiectasia macularis eruptive perstans listed from most to least common [1-4]. Here, we describe an extremely uncommon case of bullous mastocytosis in a six-month-old male infant.

## Case presentation

**Figure 1:**
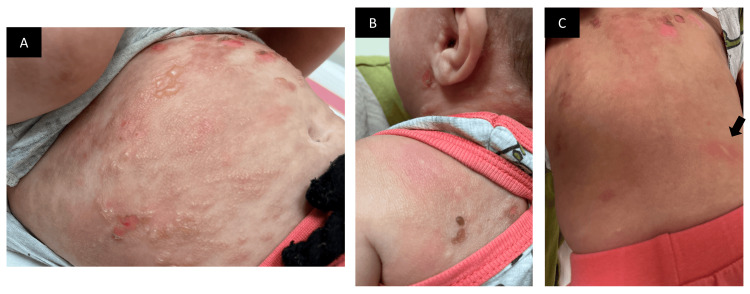
Clinical presentation. A and B: Multiple intact and eroded vesicles overlying erythematous plaques, and wheels with a peau d’orange appearance involving the abdomen and the neck/scalp area. C: Diffuse erythema and thickening of the skin on the back with a few erosions on the upper back; positive dermographism can be seen (arrow).

A six-month-old male infant born post-term by cesarean section presented to our clinic with generalized blisters on the body. The blisters started to appear one month ago. The first lesion appeared on the left elbow area and later progressed to involve the other extremities, trunk, neck, and scalp. The most affected regions were the chest and back. The lesions were associated with severe itching, especially on the back, aggravated by rubbing and bathing. The parents denied any weight loss, vomiting, diarrhea, shortness of breath, or syncope. The infant was fed formula milk only. The parents did not relate any medication use as an exacerbating factor. The last vaccination was two months ago at the age of four months. The patient had no family history of any hematologic or dermatologic disease and did not have any significant developmental, medical, drug, surgical, or social history. Physical examination of the skin showed multiple tense vesicles and bullae mainly on the trunk. The underlying skin was diffusely thickened and erythematous, having an orange peel (peau d’orange) appearance (Figures 1A, 1B). Darier sign was not demonstrated; however, dermographism was demonstrated on the back and appeared positive (Figure 1C). Palms and soles were spared. Hair, nail, mucosae, and general physical and systemic examination revealed no abnormality.

**Figure 2:**
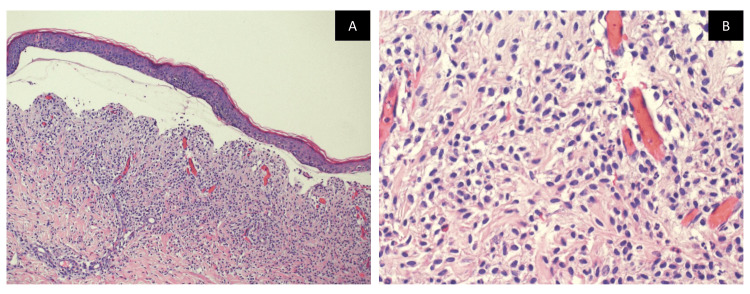
Hematoxylin and eosin stain. A: The epidermis shows a subepidermal blister. Within the dermis, there is a diffuse infiltrate composed of monotonous cells. The cells do not infiltrate the epidermis. Scattered eosinophils are noted. B: Diffuse infiltrate within the dermis composed of monotonous cells with round nuclei and granular cytoplasm, and scattered eosinophils are noted.

**Figure 3:**
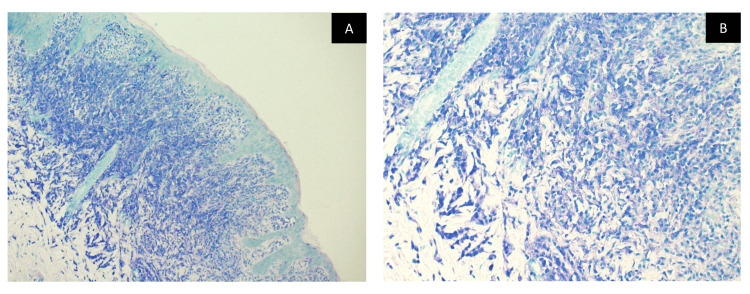
Giemsa stain. A and B: Giemsa stain confirms the increased mast cells.

**Figure 4:**
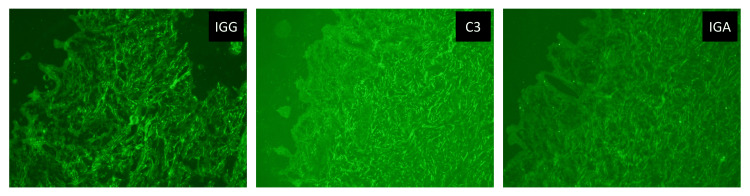
Immunofluorescence. Negative direct immunofluorescence for IgG, IgA, and C3.

Given the skin findings, our differential diagnosis included bullous mastocytosis, linear IgA bullous dermatosis, childhood bullous pemphigoid, and epidermolysis bullosa simplex, Dowling-Meara type. A complete blood cell count with differentials, C-reactive protein, liver, and renal function tests were within normal limits. Skin biopsies were obtained from three different skin lesions, including the bullous lesion, the infiltrative plaque, and the perilesional skin. Histological examination of the skin showed a subepidermal blister and a generalized infiltration of monomorphic granular cells concentrating within the papillary dermis with a few eosinophils (Figure 2). Giemsa stain was taken up by almost all infiltrating cells which confirmed the increased mast cells (Figure 3). Direct immunofluorescence was negative for IgG, IgA, and C3 which ruled out other immunobullous diseases and confirmed the diagnosis of bullous mastocytosis (Figure 4).

**Figure 5:**
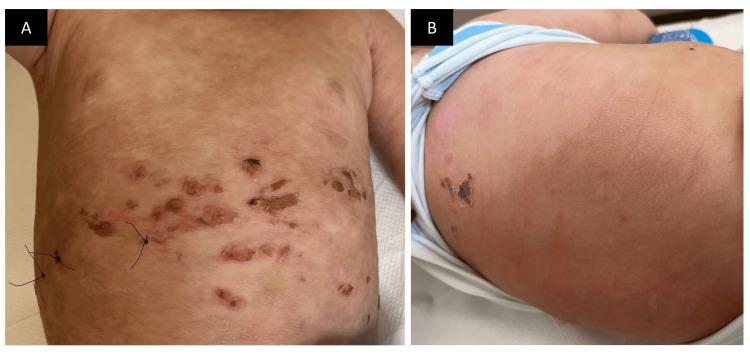
Follow-up. A and B: No new vesicles or bullae, erosions are healing, and minimal post-inflammatory hypopigmentation.

The parents were instructed to avoid all mast cell degranulation triggers such as shellfish, extremes of temperature, vigorous rubbing or friction, photo-exposure, drugs including sympathomimetics, non-steroidal anti-inflammatory drugs, narcotics, dextran, systemic anesthetics, and radiological contrast dyes. In addition, the patient was treated with cetirizine 2.5 mL syrup daily and mometasone 0.1% cream on the affected areas. A follow-up examination one week later revealed drastic improvement. The skin started to heal, the erythema reduced markedly, and no new vesicles developed (Figure 5).

Given the slightly increased risk of anaphylaxis in children with diffuse cutaneous mastocytosis, the patient was referred to pediatric hematology to prescribe an epinephrine auto-injector and exclude systemic mastocytosis. So far, the patient has been tested monthly for tryptase levels which were elevated, and the last test result was 82.2 μg/L. An abdominal ultrasound was also done which was normal.

## Discussion

Nettleship and Tay were the first to discover cutaneous mast cell disease in 1869. Nearly 100 years later, in 1963, Degos grouped the different presentations of the disease which paved the way for the current classifications of mastocytosis [1]. The prevalence of pediatric mastocytosis has been estimated to be 10 in every 100,000 people [2]. The majority of childhood cases are benign and present with the cutaneous type of mastocytosis [1,2]. Urticaria pigmentosa accounts for around 70% to 90% of the cases and the other types including mastocytomas, diffuse cutaneous mastocytosis, and telangiectasia macularis eruptive perstans account for 30% or fewer of the cases [1,2]. In our case, the patient presented with the bullous variant of diffuse cutaneous mastocytosis which is extremely rare [1-5]. It has been proposed by some researchers to have a rare occurrence of 1 in 1,000,000 individuals [3].

The vast majority of mastocytosis cases are due to a somatic mutation in the *c-kit* proto-oncogene [1-5]. *C-kit* produces the KIT receptor or CD117, a tyrosine kinase receptor that is also known as the stem cell factor receptor, and it is responsible for the differentiation, maturation, and proliferation of mast cells [1,2,4,5]. Mutations in *c-kit* produce an autoactivated KIT receptor which leads to enhanced proliferation of the mast cells and aggregation in bodily organs [1,2,4]. Familial cases of mastocytosis are seldom seen, and they are associated with germline mutations in the c-kit gene and transmitted in an autosomal dominant manner [1,2]. Some cases of mastocytosis do not show a *c-kit* mutation which implies that other genes may have a role in the development of mastocytosis [1,2].

The clinical features of bullous diffuse cutaneous mastocytosis include thickening of the skin known as pachydermia and the formation of blisters [1-5]. The blisters form from serine proteases produced by the mast cells, and the hemorrhagic element seen in some of the blisters can be attributed to heparin release [2]. Other signs include positive dermographism and Darier sign [1-5]. Some patients can develop systemic symptoms due to the release of histamine and other mast cell mediators into the blood circulation such as itching, flushing, gastrointestinal disturbance, and even anaphylaxis [1,2].

The diagnosis of cutaneous mastocytosis can be made using several clinical and biochemical tests [1,2]. Initially skin examination, systemic examination, complete blood count with differential, serum chemistry, and serum tryptase levels in addition to skin biopsy and histology are needed [1,2]. The diagnosis can be established given that the blood tests are normal, the tryptase level is below 100 μg/L, no organomegaly is present, and the skin biopsy confirmed the presence of high numbers of mast cells infiltrating the dermis [1,2,5]. Regular follow-up every six to 12 months is crucial until the resolution of the skin lesions as a small number of cases may progress to systemic mastocytosis [1,2,4].

Systemic mastocytosis is suspected in case of high serum tryptase levels, severe systemic symptoms, presence of organomegaly, no improvement with symptomatic treatment, or the persistence of the skin lesions into adulthood (as most of the cases of childhood cutaneous mastocytosis resolve around puberty) [1,2]. Bone marrow studies including bone marrow biopsy and aspirate are required for confirmation [1,2]. Systemic mastocytosis encompasses six subtypes which have a prognosis that varies from good to very poor [1,2]. Indolent systemic mastocytosis is the subtype most commonly seen in children, and, luckily, indolent systemic mastocytosis has a good prognosis [1,2].

The main goal in the treatment of cutaneous mastocytosis is to reduce and control the release of mast cell mediators [1-3,5]. The first and most important line of defense is avoidance of triggers [1-3,5]. Many triggers can lead to mast cell mediators release which can be categorized into two main groups, namely, environmental triggers and medications [1,2]. The main environmental triggers are hot and cold temperatures, fever, infections, stress, exercise, skin rubbing, allergens, and some foods such as shellfish, chocolate, tomato, pineapple, caffeine, alcohol, and synthetic food dyes and flavors [1-5]. Although medication-induced reactions are rare, it is advised to avoid them, including non-steroidal anti-inflammatory drugs, opioids, contrast media, dextromethorphan, and some antibiotics such as quinolones [1,2,4,5].

The symptomatic treatment of diffuse cutaneous mastocytosis can be achieved using topical creams and oral medications such as topical corticosteroids or topical sodium cromolyn and oral antihistamines [1-5]. H1-antagonists are utilized to manage the itching and flushing and H2-antagonists or sodium cromolyn can be added to relieve gastrointestinal symptoms [1,2,4,5]. Resistant cases can be managed with omalizumab (an anti-IgE monoclonal antibody) or using psoralen plus ultraviolet A photochemotherapy [1,2]. In the case of anaphylaxis, epinephrine intramuscular injections are crucial to save the patient’s life. In addition, omalizumab has been found to help suppress recurrent anaphylaxis episodes [1,2].

## Conclusions

Diffuse cutaneous mastocytosis is a very rare cause of bullous eruptions in children. The mainstay treatment of bullous mastocytosis includes the avoidance of triggering factors and the use of systemic antihistamines. Additionally, an epinephrine intramuscular injection is crucial in case of anaphylaxis. Most cases have a benign clinical course and resolve around puberty. This report highlights the importance of keeping diffuse cutaneous mastocytosis in the differential diagnosis of any case presenting with a bullous eruption.
